# The Role of Hydrogen Bonding in the Raman Spectral Signals of Caffeine in Aqueous Solution

**DOI:** 10.3390/molecules29133035

**Published:** 2024-06-26

**Authors:** Sara Gómez, Chiara Cappelli

**Affiliations:** Scuola Normale Superiore, Classe di Scienze, Piazza dei Cavalieri 7, 56126 Pisa, Italy

**Keywords:** Raman spectroscopy, Resonance Raman, caffeine, simulations, Molecular Dynamics, hydrogen bonding, UV-Vis, Quantum Mechanics/Fluctuating Charges, non-covalent interactions

## Abstract

The identification and quantification of caffeine is a common need in the food and pharmaceutical industries and lately also in the field of environmental science. For that purpose, Raman spectroscopy has been used as an analytical technique, but the interpretation of the spectra requires reliable and accurate computational protocols, especially as regards the Resonance Raman (RR) variant. Herein, caffeine solutions are sampled using Molecular Dynamics simulations. Upon quantification of the strength of the non-covalent intermolecular interactions such as hydrogen bonding between caffeine and water, UV-Vis, Raman, and RR spectra are computed. The results provide general insights into the hydrogen bonding role in mediating the Raman spectral signals of caffeine in aqueous solution. Also, by analyzing the dependence of RR enhancement on the absorption spectrum of caffeine, it is proposed that the sensitivity of the RR technique could be exploited at excitation wavelengths moderately far from 266 nm, yet achieving very low detection limits in the quantification caffeine content.

## 1. Introduction

Caffeine (1,3,7-trimethylxanthine) is a purine alkaloid that acts as a psychoactive drug and stimulant agent [1]. It can be consumed from natural sources like coffee, chocolate, and tea, but it can be found in other foods and beverages, and, in recent decades, also in medications, because of the enhanced effect it provides in a mix with certain analgesics [2]. The effects of caffeine on cognitive or physical performance have been the subject of several reviews [3,4,5,6].

**Figure 1:**
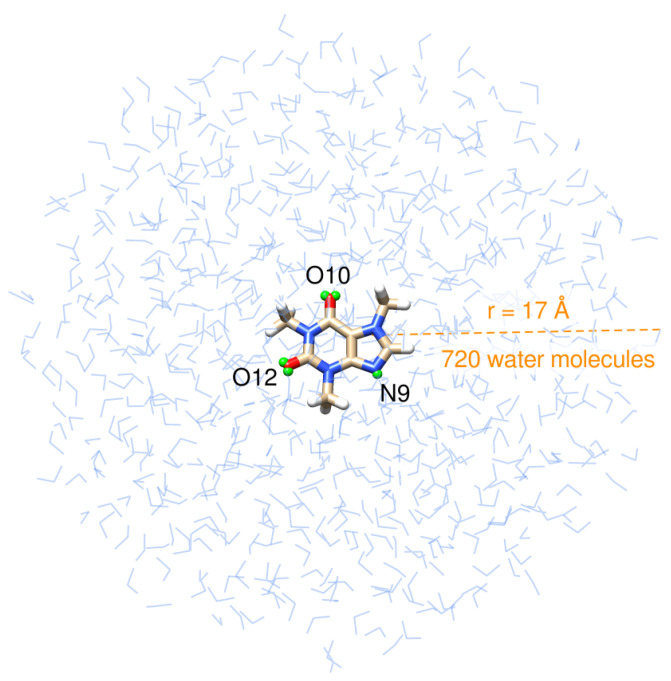
Pictorial view of caffeine dissolved in aqueous solution as treated in QM/FQ calculations. Virtual sites for the atoms that are more prone to hydrogen bonding with the solvent, labeled N9, O10, and O12, are shown in small green spheres.

Structurally, caffeine is a combination of two fused rings: pyrimidinedione, a six-membered pyrimidine ring with two ketone groups linked to it at meta positions, and imidazole, a five-membered ring with two nitrogen atoms. Caffeine’s structure is shown in the innermost layer of the drop in Figure 1.

Due to the high level of caffeine consumption worldwide, controlling the caffeine content of coffee, tea samples, and caffeine-containing food products is indispensable [7]. Moreover, this methyl xanthine, as well as diclofenac, ibuprofen, and acetaminophen, is currently considered an emerging contaminant that poses health risks to aquatic life [8] and poses a danger to the environment because of its persistence [9,10]. In fact, caffeine and paraxanthine pollution in aqueous environments are declared to be ubiquitous [11]. Hence, the determination of caffeine is relevant in chemical analysis and it is desirable to have practical methods for carrying it out in various matrices. Many analytical techniques have been employed for that goal, among them Raman spectroscopy [7,12]. As a matter of fact, the application of vibrational spectroscopy techniques to quantify nutraceuticals in fruits and plants has proven successful [13], and Raman spectroscopy has been used for quantitative analysis in the pharmaceutical industry [14,15].

The evolution of Raman as an extremely sensitive analytical tool was further boosted by the discovery of several other Raman phenomena, including Resonance Raman (RR), coherent anti-Stokes Raman scattering (CARS), and surface-enhanced Raman scattering (SERS) [16,17]. All of them can improve the intensity of the Raman signal, thus overcoming the low sensitivity caused by the inherently weak Raman scattering, and in most cases, they can minimize the interference from fluorescence. In contrast to SERS, which is a method where the sample is adsorbed onto a metallic substrate (gold, silver, etc.) [18], RR benefits from choosing an excitation wavelength that couples to an electronic transition or photon absorption of the target analytes and chromophore segments of material or macromolecules, affording a much more intense Raman spectrum. Thus, some modes absent in normal Raman spectra can be resonantly enhanced to appear in RR spectra. The combination of the two techniques, RR and SERS, gives rise to surface resonance-enhanced Raman spectroscopy (SERRS) and can greatly increase sensitivity by up to 10 orders of magnitude compared to just using RR [7]. In the context of determining caffeine content, the low solubility of the compound in water at room temperature has been a limitation to employing conventional Raman methods; nonetheless, it has been investigated with the SERS modality in various works [19,20]. SERS has afforded a simultaneous multiplexed quantification of caffeine and its major metabolites theobromine and paraxanthine [21], and SERS-coupled multivariate calibration has been recently used in the rapid prediction of caffeine in tea [22]. In addition, Frosch et al. [23] presented a fiber-array-based Raman hyperspectral imaging technique for direct simultaneous in situ monitoring of different active pharmaceutical ingredients, such as caffeine, in analgesic tablets. Without requiring metallic surfaces or further agents, diluents, or matrices, RR represents an excellent technique for single or simultaneous identification and quantification of multiple components of interest in a sample thanks to its good selectivity and sensitivity [24,25,26,27,28]. To our knowledge, there is only one experimental study in which RR has been used for the caffeine spectral analysis of extremely diluted samples, such as 0.0022 M [29].

Quantum chemical calculations are useful tools to properly describe and analyze Raman and in particular RR spectra [30,31,32,33,34,35]. Some modeling with isolated caffeine in the gas phase or describing the condensed phase using the Polarizable Continuum Model (PCM) [36] or a microsolvation approach has been used in several works to assist the peak assignments of Raman spectra [19,29,37,38,39,40,41,42]. Nevertheless, understanding the properties and spectral behavior of caffeine in solution requires a computational strategy that includes conformational sampling, environmental effects with a proper description of hydrogen bonding (HB) interactions, the dynamical aspects of the solvation, and mutual solute–solvent polarization [43]. Such features are all integrated into Quantum Mechanics/Fluctuating Charges (QM/FQ) computational protocols developed during the last years in our research group [44,45,46]. By following the protocol, we have previously studied the absorption spectra of caffeine and similar xanthines [47,48] and so far we have also computed RR spectroscopies on solvated amides [49], dipeptides [50], anionic ibuprofen [51], contaminants [52], doxorubicin (DOX) and DOX-DNA [53] complexes. As a general finding, we have seen that the multiscale QM/FQ protocols outperform implicit and non-polarizable QM/Molecular Mechanics approaches, and this is due to the atomistic treatment of the solvent molecules and thus of the solute–solvent interactions. On the subject of intermolecular interactions, Natural Bond Orbitals (NBO) [54,55,56] and the topological analysis of electron density as formulated in the Quantum Theory of Atoms in Molecules (QTAIM) by Bader [57] are well-established tools for studying these cooperative, non-covalent contacts and have been applied to diverse chemical systems [58].

The purpose of this paper is to use computational tools to analyze intermolecular interactions and provide an overview of the chemical physics influence of the environment when recognizing resonance enhancement in the Raman spectrum of caffeine in an aqueous solution. Since the careful selection of a laser source that is appropriate for a specific compound and the geometry of the spectrometer is essential to the efficient use of RR spectroscopy as an analytical tool [59], several excitation wavelengths are proposed to quantify the caffeine content in very diluted samples.

The next section outlines the computational steps, while the modeled electronic absorption, Raman, and RR spectral profiles for solvated caffeine are then discussed in light of the solute–solvent interactions.

## 2. Results and Discussion

This section provides a characterization of the main interactions taking place when caffeine is dissolved in water as well as a description of the multiscale QM/FQ simulated electronic absorption, Raman, and RR spectra, whose quality and interpretation are improved by an atomistic description of the environment in the modeling.

Geometrical and energetic aspects of isolated and hydrated forms of caffeine have been the subject of investigation in earlier studies [38,40,60,61,62]. Structural analysis of caffeine has revealed small barriers associated with the rotation of the methyl groups [47] which are reported to be affected by non-covalent interaction with their neighboring functional groups and by different degrees of hyperconjugation [39,63]. Despite its limited solubility in water that is certainly overcome at moderate temperatures, caffeine solutions can be seen as a series of non-covalent interactions, mainly HBs.

### 2.1. Caffeine⋯Water Interactions

**Figure 2:**
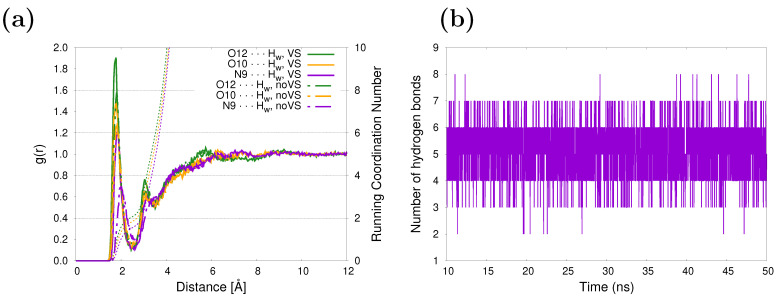
(**a**) Radial distribution function between selected sites of caffeine and water molecules. Running coordination numbers are also included (dashed lines). (**b**) Evolution in time of the number of hydrogen bonds between caffeine and its surrounding water molecules.

The hydrogen-bonding patterns of caffeine in an aqueous solution may be examined from the MD trajectories. Caffeine has three specific solvation sites and five hydrogen bond acceptor sites: four acceptor sites are provided by the carbonyl oxygen atoms and one by a nitrogen atom in the imidazole ring. The solvent structure around those atoms is shown in Figure 2a. The two carbonyl oxygen atoms, O10 and O12, are better solvated than the N9 site, as reflected by the higher peak value in the RDFs associated with the former. The corresponding running coordination numbers (RCNs) resulting from integrating the RDFs over the first solvation shell give approximately 3.9 water molecules around the carbonyl oxygens and 1.6 for the nitrogen, yielding a total of 5.5 water molecules close to caffeine. A similar number was obtained in our previous work [47], with the difference being in the incorporation of virtual sites (see Figure 1) in the lone pairs of O10, O12, and N9. Here, it is clear that as a consequence of a more specific/directional interaction when adding virtual sites, the peaks of the RDFs are higher and shifted to smaller distances (continuous vs. dashed curves in Figure 2a). After studying the influence of HB directionality on vibrational cooling dynamics of methyl xanthines, Zhang et al. [64] suggested that HB acceptors do not play a primary role in mediating vibrational energy flow from solute to solvent. Unsurprisingly, the five water molecules in the first solvation shell coincide with the average number of HBs between the caffeine and the solvent based on geometric criteria ($d_{X\cdots O_{w}}\leq 3.5$ Å and $\theta_{H_{w}-O_{w}-X}\leq 30{}^{\circ}$, X being N9, O10, and O12 atoms in caffeine), as can be inferred from Figure 2b. The hydration structure of caffeine in aqueous solution has been studied in earlier [65] and recent [66] works using classical MD simulations, and the same hydration patterns were found.

To further pinpoint the source of the stabilization when caffeine is dissolved in an aqueous solution, more selective scrutiny is included by performing a quantitative analysis of intermolecular interactions with NBO and QTAIM approaches. Given that determining explicit interactions of caffeine atoms with environmental molecules requires the explicit presence of water molecules, the QM region was expanded on each snapshot to accommodate the first solvation shell in addition to caffeine, but still keeping the other FQ water molecules. First, we resort to NBO to study NBO donor–acceptor interactions.

**Figure 3:**
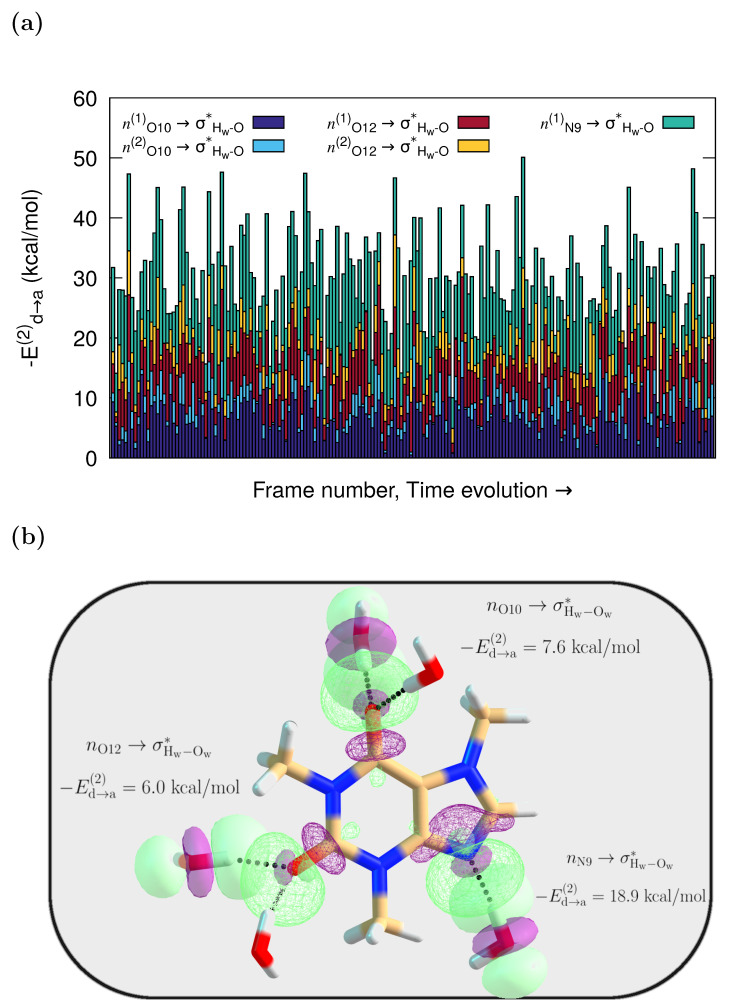
QM/FQ NBO stabilization energies. (**a**) Cumulative quantification of the strength of the orbital interactions that keep caffeine in contact with adjacent water molecules. All stabilization energies, $E_{d\to a}^{\left(2\right)}$, are associated with the $n_{O_{c}}\to \sigma_{H_{w}-O}^{*}$$O_{c}=O10,O12$ charge transfers, and $n_{N9}\to \sigma_{H_{w}-O}^{*}$ charge transfer. (**b**) Orbital representation within the NBO picture for the intermolecular interactions in solvated caffeine for one randomly chosen configuration from the MD trajectory. The QM region includes five explicit waters for that configuration, while the remaining solvent molecules are treated at the FQ level.

According to the NBO results, caffeine and its neighboring water molecules interact through delocalizations from lone pairs (*n*) in the solvent to antibonding $\pi^{*}$ orbitals in the solute, or from π Lewis type orbitals in caffeine to antibonding $\sigma_{H_{w}-O_{w}}^{*}$ in the water molecules. However, the dominant specific solute–solvent contacts involve HBs with orbital interactions from lone pairs (*n*) in the O10, O12, and N9 caffeine atoms to antibonding ($\sigma^{*}$) orbitals in the water molecules. They are of the $n_{O_{c}}\to \sigma_{H_{w}-O}^{*}$$O_{c}=O10,O12$, $n_{N9}\to \sigma_{H_{w}-O}^{*}$ type. Their interaction energies on each frame, obtained via second-order perturbation corrections to the Fock matrix, are presented as stacked histograms in Figure 3a. The corresponding orbital overlap representation for one of the MD configurations where caffeine more heavily interacts with five water molecules is depicted in Figure 3b.

The average values of stabilization energies in Figure 3 are around $-E_{d\to a}^{\left(2\right)}$ = 7 kcal/mol and $-E_{d\to a}^{\left(2\right)}$ = 11 kcal/mol, for $n_{O_{c}}\to \sigma_{H_{w}-O}^{*}$$O_{c}=O10,O12$, $n_{N9}\to \sigma_{H_{w}-O}^{*}$, respectively, which are associated with moderate or mild interactions, unlike the strong interaction found for charge-assisted HBs [51,67]. As is also true for the reported UV-Vis and ECD spectra of captopril and naproxen [67], configurations with a large cumulative $|E_{d\to a}^{\left(2\right)}|$ have spectra that better match the experiments. The individual stabilization that those charge transfers provide to the system is quantified in Figure S1 in the Supplementary Materials.

Two observations can be made from the cumulative plot in Figure 3a. First, the different interaction strengths seen for each snapshot underline the variability in the solvent arrangements around the caffeine molecule. This, in turn, directly affects the spectra because as a result of such dynamical variability, assorted oscillator strengths and Raman cross-sections are obtained for each snapshot. Second, given that the higher the $|E_{d\to a}^{\left(2\right)}|$ value, the stronger the interaction, the water contacts with the lone pair in the N9 atom are the strongest ones most of the time ($-E_{d\to a}^{\left(2\right)}$ up to 26 kcal/mol, Figure S1), even if all the interactions have the same nature. Interestingly, in the crystal structure of caffeine hydrate, the only existing hydrogen bond between the crystallization water and the caffeine molecule is noted to occur through the hydrophilic N9 center [68].

**Figure 4:**
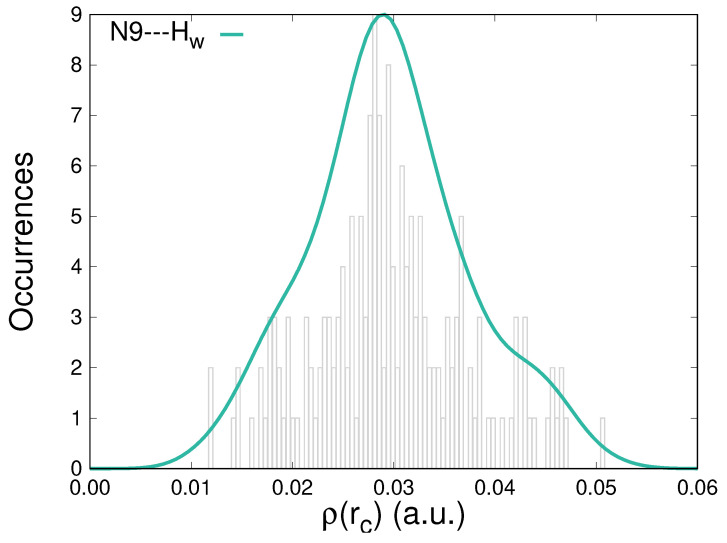
Distribution of the electron densities at the bond critical points for the *N*9⋯*H_w_* intermolecular contacts between caffeine and water molecules along an MD trajectory.

Further characterization of the hydration patterns of solvated caffeine is carried out by analyzing the topological descriptors, particularly the electron density, $\rho (r_{c})$, accumulated at the BCPs that correspond to HBs. Figure 4 displays the distribution of the $\rho (r_{c})$ values for located $N9\cdots H_{w}$ contacts in the whole group of configurations. Electron densities at those points cover the [1.0 × 10^−2^, 5.0 × 10^−2^] a.u. interval. Similar to what happens for the interaction with the other atoms susceptible to H-bonding in caffeine (Figure S2 in the Supplementary Materials), the distributions are centered around 3.0 × 10^−2^ a.u., thus exhibiting larger densities than the usual reference of the water dimer, 2.3 × 10^−2^ a.u. [69]. For the interactions studied in this work, the accumulations of electron densities at the BCPs do not present any direct correlation with the $-E_{d\to a}^{\left(2\right)}$ stabilization energies.

### 2.2. Spectra

After sampling the caffeine⋯water phase space through MD simulations and characterizing the dynamic nature of their interactions, this section reports on the simulated UV-Vis, Raman, and RR spectra of solvated caffeine, making a comparison with experimental data and offering a detailed view of the enhanced signals.

Convergence tests displayed in Figures S9–S11 in the Supplementary Materials indicate that UV-Vis, Raman, and RR spectra remain unchanged when more than ≈100 uncorrelated snapshots are included. Next, the results and discussion built upon converged spectra averaged on 200 frames in each case. We recall here that for all of the spectral calculations, the caffeine moiety is the only one treated at the QM level, whereas all the water molecules are described with FQ.

#### 2.2.1. UV-Vis Spectrum

**Figure 5:**
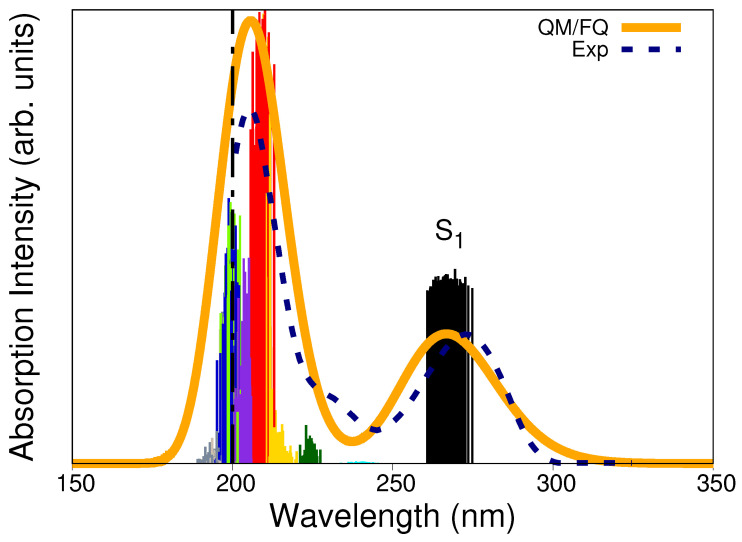
QM/FQ and experimental UV–Vis absorption spectra of caffeine in aqueous solution. Ten excited states, each one associated with a different stick color, were converged in the TD-DFT calculations. The spectrum was convoluted with Gaussian functions using a FWHM of 0.6 eV. The label $S_{1}$ indicates the first excited states. Experimental reports collected from refs. [70,71] determine the first maximum at 273 nm. The dashed vertical line indicates that there is no experimental information below 200 nm.

The QM/FQ calculated absorption spectrum of solvated caffeine is shown in Figure 5 along with the experimental curve (dashed line) and the stick-like spectrum colored according to every specific excited state. Two main bands characterize the UV-Vis absorption spectrum. Excitations to $S_{1}$ (black sticks) are mainly responsible for the appearance of the band centered at around $\lambda_{max,1}=$ 267 nm. In contrast, the band with the highest absorption is located at $\lambda_{max,2}=$ 206 nm and results from a more complex combination of excitations. Experimental observations placed such bands at 273 nm and 205 nm, respectively, [70,71] or at very similar values [38,39,61,72]. We have previously concluded [47] that the vertical absorption in the lowest energy band corresponds to a $\pi \to \pi^{*}$ electronic transition involving the HOMO and LUMO orbitals, based on the canonical molecular orbital decomposition [56]. In line with that assignment, Table S1 in the Supplementary Materials reports, for a single snapshot, the nature of the electronic states giving rise to the absorption spectrum. Beyond the fair agreement with the experimental spectrum, the computed UV-Vis absorption spectrum helps recognize potential excitation wavelengths for irradiating a solvated caffeine sample in RR spectroscopy (see below).

Multiscale methodologies have also been employed by Skarmoutsos et al. [66] to calculate absorption and emission spectra of caffeine in aqueous solution and the authors emphasized the need for an explicit inclusion of the solvent for the correct reproduction of the emission spectrum.

#### 2.2.2. Spontaneous Raman and UV Resonance Raman Spectra

Despite having noticed in our previous publication that specific solute–solvent interactions are not essential for the reproduction of excitation energies of caffeine in aqueous solution, they are found to be significant in the modeling of relative intensities of the two main bands of the spectrum [47] and become fundamental when going to vibrational spectroscopies as we have already exemplified for amides [49], dipeptides [50], and anionic ibuprofen [51] cases.

Multiple works in the literature deal with the Raman spectra of caffeine at different excitation sources. Some of the experiments refer to the hydrated and anhydrous crystalline caffeine [42,68,73,74], whereas others cover caffeine solutions [29,75] and the effect of different pH values on the Raman spectra [19,42]. Calculations have also been used as guidelines for a reliable and complete vibrational assignment [19,40,42,60,76,77]. Since caffeine does not have tautomers in water, only small changes are appreciated in the peak assignments for the crystalline and solvated forms. The most relevant vibrational frequencies in the 1000–2000 cm^−1^ range of the caffeine Raman spectra are ascribed to the following:

- Carbonyl stretching frequencies, $\nu_{C=O}$, experimentally reported to appear at 1647 and 1692 cm^−1^ (theoretically located at 1728 and 1670 cm^−1^, respectively) and due to the two C=O groups couple into an in-phase and an out-of-phase stretching vibration. The in-phase one is predicted to have less intensity (sometimes a half) than the out-of-phase carbonyl vibration [40].
- C=C and C=N stretching modes in the purine ring system, $\nu_{C=C}$ and $\nu_{C=N}$, at 1598 cm^−1^.
- Imidazole ring stretching plus $\nu_{C-N}$ + $\nu_{C=C}$ + C-H bend appearing as a peak at 1549 cm^−1^.
- Symmetric CH-bending vibrations in the methyl groups appearing as a broad band or a set of peaks [40] at 1488, 1470, 1454, and 1431 cm^−1^. These are collectively labeled as $\delta_{\mathrm{CM}}$ in ref. [29] and centered at approximately 1497 cm^−1^. Importantly, some of them also include a contribution of the $\nu_{C-N}$ in the imidazole ring.
- Bending of C8-H atoms in the imidazole group combined with CH_3_ bending of methyl groups, located at about 1437 cm^−1^. This peak is labeled as $\delta_{C8-H}$ in ref. [29].
- Joint quadratal $\nu_{\left(\mathrm{imidazole}\mathrm{ring}\right)}$, $\nu_{C=N}$ and bending of methyl groups at 1406 cm^−1^.
- Stretching in the imidazole ring $\nu_{C-N}$ plus some bending of methyl groups at 1362 cm^−1^. It will be called $\nu_{i-\mathrm{ring},1}$ in what follows.
- Trigonal C-N stretching vibrations in the imidazole ring combined with N-CH_3_ (M1) stretching vibration at about 1335 cm^−1^. In some reports, this is the most intense Raman peak and is labeled as νi−ring in ref. [29]. It will be termed as $\nu_{i-\mathrm{ring},2}$ in the next discussion.
- Mixed $\nu_{N-{\mathrm{CH}}_{3}\left(M2\right)}$, $\nu_{C-N}$ and $\nu_{C-C}$ in both rings expressed as a band centered at 1291 cm^−1^. The general $\nu_{\mathrm{rings}}$ nomenclature proposed in ref. [29] for this peak will be maintained here.
- CH_3_ rocking (in plane), $\rho_{{\mathrm{CH}}_{3},M1}$ and $\rho_{{\mathrm{CH}}_{3},M3}$ located at 1034 and 1079 cm^−1^, respectively.

**Figure 6:**
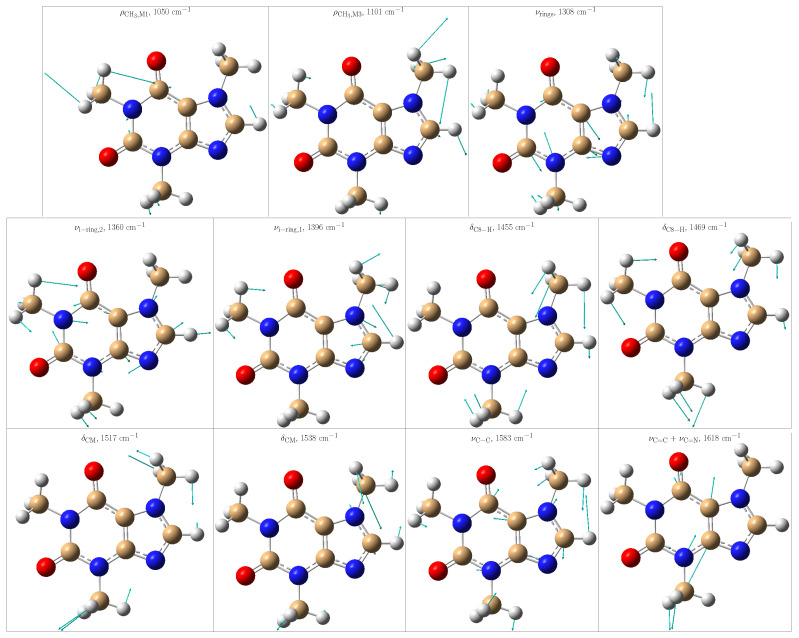
Selected vibrational modes giving rise to the most enhanced peaks in the Resonance Raman spectrum of caffeine in aqueous solution. Color code: C atoms in tan, O atoms in red, N atoms in blue, and H atoms in light gray.

The theoretical positions of those peaks in one specific frame and after averaging the convoluted spectra along all MD snapshots are listed in Table 1. From the standard deviations, it is noticed that the positions of some peaks are particularly sensitive to the solvent arrangements, thus indicating either close contact with the solvent or limited vs. more flexible vibrations due to the presence/absence of water molecules in the vicinities. To offer a better description of the normal modes, Figure 6 depicts some selected ones.

**Figure 7:**
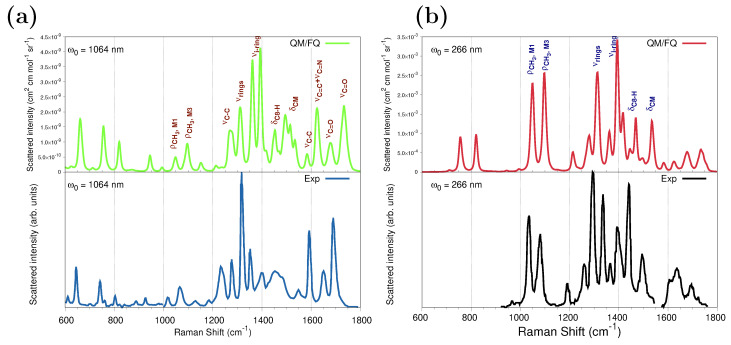
Convoluted QM/FQ (top) and experimental (bottom) Raman spectra of caffeine in aqueous solution. (**a**) Spontaneous (far from Resonance) Raman spectra simulated and experimental measurement from ref. [68] using 1064 nm as excitation wavelength. (**b**) UVRR spectra. RR intensities were calculated with a 200 cm^−1^ damping factor. In the UVRR experimental spectrum [29] (bottom panel), the authors reported that data at about 1550 cm^−1^ are affected by an experimental laser satellite artifact and therefore were omitted. Raman and RR intensities were broadened using Lorentzian functions with FWHM = 8 cm^−1^. No scaling factors were applied to the frequencies. See also Figure S1a for atom numbering.

Figure 7a shows the QM/FQ spontaneous (far from Resonance) Raman and the experimental spectra [68] at $\omega_{0}=$ 1064 nm. We have chosen data from ref. [68] as a reference because of the larger measurement region available to compare, but spectral features are common to all experimental works. The calculated frequencies agree very well with the experimental ones, and the computed intensities accurately capture the relative intensities of the main peaks. Consistent with previous findings, the configurational wealth translated in a diversity of sticks (Figure S5a in the Supplementary Materials) confers the modeled spectrum with a natural inhomogeneous broadening that brings it close to the measurements.

To investigate the RR enhancement pattern, Figure 7b compares the computed QM/FQ RR spectrum of caffeine aqueous solution with UVRR scattering measurements reported in ref. [29] and acquired at $\omega_{0}=$ 266 nm. Figure S5b in the Supplementary Materials depicts raw data plotted as a stick-like RR spectrum. The convoluted RR spectrum follows a similar pattern as that seen in the experiment and most experimental relative intensities are well reproduced. The position of one of the computed absorption maxima in Figure 5 matches the experimental RR condition; thus, simulating at 266 nm preserves the resonance enhancement observed in the experiment. Under this excitation, of particular spectroscopic interest are changes involving $\rho_{{\mathrm{CH}}_{3},M1}$, $\rho_{{\mathrm{CH}}_{3},M3}$, $\nu_{\mathrm{rings}}$, $\nu_{i-\mathrm{ring},2}$, $\delta_{C8-H}$ and $\delta_{\mathrm{CM}}$ signals. This result agrees with the earlier experimental RR studies of caffeine in solution by Tavagnacco et al. [29]. The authors pointed out that this quite unselective peak enhancement obeys the fact that the orbitals mainly contributing to the transition at 273 nm are highly delocalized along the entire molecule. The Raman and RR intensities for those vibrations are summarized in Table 1, for calculations performed at 1064 nm and 266 nm, respectively, and the enhancement goes up to six orders of magnitude for some vibrations.

It was mentioned before that the strongest absorption band of solvated caffeine occurs at 267 nm (273 nm in the experiments) and is merely owing to the contribution of the $S_{1}$ excited state. Calculated Raman spectra at a 266 nm excitation wavelength using the ten excited states and only $S_{1}$ are shown in Figure S6a of the Supplementary Materials and are virtually identical. Instead, using excited state gradients for states going from $S_{2}$ (sticks in cyan in Figure 5) to $S_{10}$ (sticks in gray in Figure 5), which are the cause of the appearance of the other more intense band (at 205 nm), produces an RR spectrum with weaker signals and introduces new spectral features.

As is to be expected, the RR enhancement of some normal modes of caffeine in water is highly dependent on the excitation wavelength. Based upon the results of the UV-Vis spectra in Figure 5, the effect of using excitation wavelengths near the center of the highest absorption band on the RR spectrum was also explored. As illustrated in Figure S6b of the Supplementary Materials, choosing $\omega_{0}=$210 nm leads to a quite different spectral profile that alters the intensity of the peaks revealing another enhancement pattern when compared against the spectrum at $\omega_{0}=$ 266 nm. In that scenario, Raman signals around 1600 cm^−1^, $\nu_{C=C}$, $\nu_{C=N}$, and $\nu_{C-C}$, as well as $\nu_{i-\mathrm{ring},1}$ appear to be substantially enhanced. Enhancement factors at 210 nm are tabulated in Table 1 and indicate that it is possible to track the presence of caffeine by analyzing further signals that are selectively enhanced at shorter wavelengths.

The observed signal enhancement in the RR spectrum can also be rationalized in terms of the displacement of the excited states and $S_{i}$ geometries with respect to the ground state. In the (VG|FC) approximation, such displacements are related to the shift vectors (widely denoted as **K**) since the Duschinsky rotation is ignored. As noted in Figure 5, $S_{1}$ dominantly contributes to the resonance when exciting caffeine in water at 266 nm. For that particular excited state, a graphical representation of the shift vector, and in turn, of the displacements, $\Delta_{1}$ obtained by projecting the excited state gradient onto the normal modes is reported in Figure S7 in the Supplementary Materials for one of the snapshots extracted from the trajectory. Noticeably, the most intense peaks in the RR spectrum (see Table 1 for their positions) are associated with the normal modes exhibiting the largest shifts/displacement values which is exactly the spirit of the short-time approximation, although that one is better suited for the pre-resonance conditions [78,79].

**Figure 8:**
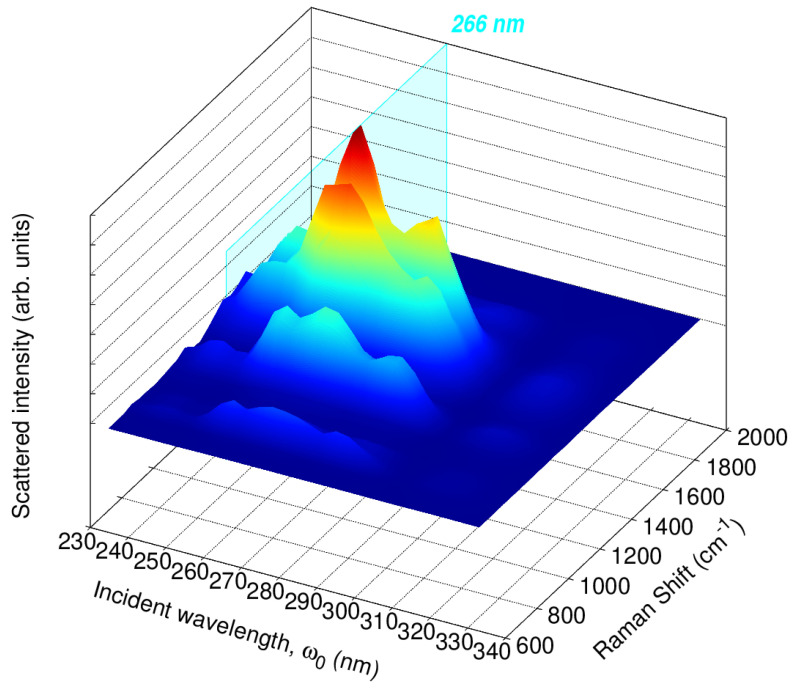
Calculated QM/FQ Resonance Raman Excitation Profiles (RREP) of caffeine in aqueous solution. In total, 200 structures were considered to achieve convergence at the QM/FQ level, with B3LYP/6–311++G(d,p).

To further investigate the relationship between the RR enhancement and the absorption spectrum of caffeine, we split the 65,000–30,000 cm^−1^ range (154–333 nm) into 15 excitation wavelengths and computed RR cross-sections. The resulting RREP is shown in Figure S8 in the Supplementary Materials. Figure 8 displays an RREP portion for excitation wavelengths in the vicinity of 266 nm, namely, 222, 235, 250, 267, 285, 307, and 333 nm, which are all associated with the lowest energy band of the absorption spectra in Figure 5. RREPs support the observation that the strongest Raman signals suggested to detect or quantify caffeine come from enhancements with $\omega_{0}$ values around 210 and 266 nm.

## 3. Materials and Methods

To analyze the potential effect of the explicit water molecules on the spectra of solvated caffeine, we initially computed all the spectra of interest both in the gas phase and in a mimicked bulk solvent environment with the implicit model PCM. Results for Raman and RR are shown in Figure S4 in the Supplementary Materials. Although the absorption spectrum is less sensitive, vibrational spectra exhibit erroneous positions of the peaks and overestimate the enhancement of some signals when compared against experiments. Due to the discrepancies between the QM/PCM and the Raman and RR experimental data, we opted to utilize an atomistic representation of the solvent in what follows.

There is a robust computational protocol, described by Giovannini et al. [44], to compute spectra of molecular systems. Such a methodology relies on a fully atomistic and polarizable classical modeling of the solvent coupled with a QM description of the solute. Recently, it was successfully applied to simulate the UV-Vis spectra of a series of methylated xanthines [47], including caffeine, in an aqueous solution. Under the assumption that virtual sites could recover the correct directionality of hydrogen bonds between the solute and solvent [49,80,81,82,83], we followed the same procedure outlined in ref. [47] but placed virtual sites at the nitrogen atom, N9, of the imidazole ring, and at the oxygen atoms, O10, and O12, of the pyrimidinedione ring, specifically at their centroid positions, determined by the Boys localization procedure [84]. Figure 1 depicts the position of those dummy atoms. MD simulations were performed in the GROMACS software, version 2020.3. Ref. [85] using the General Amber Force Field (GAFF) [86] and TIP3P [87] force fields to describe inter and intra-molecular interactions for caffeine and water molecules, respectively. From the 50 ns trajectory, we obtain the average number of solute–solvent HBs and extract the radial distribution functions (RDFs) between water molecules and the carboxylic groups and the nitrogen atom, N9, of the imidazole ring of caffeine, using the TRAVIS package [88,89]. We also determine the number of waters in the first solvation shell by integrating the RDFs.

After extracting 200 uncorrelated snapshots from the trajectory and cutting them into a solute-centered sphere with a radius of 17 Å (Figure 1), QM/FQ calculations were carried out at the B3LYP/6-311++G(*d*, *p*)/FQ level in the Gaussian16 package [90] exploiting the FQ parametrization proposed by Rick et al. [91]. The same QM level of theory has been employed in other computational works concerning caffeine and xanthines [38,39,40,47,61,63,70,77]. In spectral calculations, the caffeine molecule is the only part of the system treated at the QM level. However, we expanded the QM region to include the solvent molecules in the first solvation shell to estimate the strength of the interactions between caffeine and water molecules in the closest contact. This is carried out using the NBO analysis [54,55,56] in the NBO7 program [92]. Similar QM/FQ NBO analyses have been conducted for common pharmaceuticals [51,67] and hypoxanthine [48] in solution. Conventional bond critical points (BCP) of the electron density and localized Natural BCPs topological descriptors are computed with the Natural BCP (NBCP) analysis as implemented in NBO7 [92].

The caffeine geometry was optimized in each snapshot at the QM/FQ level by keeping the solvent molecules fixed. Vertical excitation energies were then estimated by converging 10 excited states again exploiting the B3LYP/6-311++G(*d*, *p*)/FQ level in the linear response time-dependent density functional theory (TD-DFT) framework. As a side note, optimizing the solute, or including explicit water molecules during the TD-DFT calculation did not have any effect on the UV-Vis absorption spectrum, as can be seen in Figure S3 in the Supplementary Material (SM). The reported averaged absorption spectrum was obtained by convoluting peak intensities with Gaussian functions and a full width at half maximum (FWHM) of 0.6 eV.

On the optimized geometries, frequencies and excited state gradients were also computed to model vibrational spectroscopies. Using analytical response theory as implemented for QM/FQ [93], the spontaneous Raman spectrum was calculated in the dynamic regime by setting the incident frequency ($\omega_{0}$) to match the experimental value of 1064 nm. We used the QM/FQ approach adapted to RR spectroscopy in ref. [49] and computed the RR cross-sections by resorting to the time-independent sum-over-state formulation detailed in refs. [79,94]. Among the diverse ways for modeling the excited-state Potential Energy Surface, we chose the Vertical Gradient, Franck–Condon (VG|FC) approximation, which assumes that the vibrational frequencies and normal modes of the excited state are the same as those of the ground state, while the transition dipole moments are thought to be unaffected by the geometry of the molecules. An RR excitation profile (RREP) was also built by choosing several incident wavelengths and recomputing RR cross-sections. Sticks for spontaneous Raman and RR were convoluted with Lorentzian profiles and FWHM values of 8 cm^−1^. Final UV-Vis, Raman, and RR spectra were obtained by averaging over the spectra of the MD snapshots. The convergence of QM/MM computed spectra was closely examined by considering increasing numbers of snapshots (see Figures S9–S11 in the Supplementary Materials).

## 4. Conclusions

In this study, interactions and spectral signatures of caffeine in aqueous solution have been investigated from a computational perspective to understand the impact of the dynamical variability of the solute–solvent interactions on modeled spectroscopies.

The configurational landscape of neutral caffeine in water was explored with MD simulations. From the trajectory, the hydrogen bond formation between the caffeine and surrounding waters was the dominating intermolecular interaction. The non-covalent interactions in the caffeine–water complexes were thoroughly examined using NBO and QTAIM. Among the three H-bonding sites, the caffeine interactions through the nitrogen atom of the imidazole ring turned out to be stronger than those with the two C=O groups.

Building on the fact that several analytical methods—including UV-Vis and Raman—have been proposed to determine the caffeine content in coffee samples or to detect if this emerging and persistent micropollutant is present in the environment even at very low concentrations, electronic absorption and mixed electronic–vibrational spectra were also modeled. Computed spectral profiles obtained with a multiscale QM/FQ approach were compared with experimental UV-Vis, Raman, and RR data of solvated caffeine, and a good agreement was found between simulated and experimental results. The quality of the spectra upon atomistically treating the solvent confirmed that HB interactions play an essential role in the caffeine spectral behavior. This underscores the robustness of a computational protocol able to prioritize the explicit intermolecular interactions [62] and the dynamical aspect of solvation, both important characteristics to be considered in the reproduction of any spectra, but crucial when the presence of solvent molecules influences the vibrational modes as also seen in the case of the RR spectrum of cytosine in water [35]. For caffeine, these modes are the carbonyl stretchings and C=N stretchings in the imidazole ring, the latter being involved in one of the enhanced vibrations.

From the RREPs, the excitation wavelengths found to provide an intense enhancement are those that match the maxima in the absorption spectrum, namely the close vicinity of 205 and 273 nm, mostly enhancing $\nu_{i-\mathrm{ring}}$ and $\nu_{C=C}$ signals with enhancement factors up to six orders of magnitude when compared to their intensities in conventional Raman. These results suggest that RR can be employed as a quantitative technique regarding caffeine. Indeed, experiments to monitor vibrational modes at specific excitation wavelengths, shorter than the earlier tested $\omega_{0}$ = 266 nm, can be useful to more thoroughly detect and quantify caffeine in different samples, which is a growing need because of the everyday use of this stimulating agent.

## Figures and Tables

**Table 1:** Raman and RR cross-sections in cm^2^ cm mol^−1^ sr^−1^ for selected vibrations of solvated caffeine. Two peak positions are given: in one specific frame and after averaging the spectra along all MD snapshots. StdDev stands for the standard deviation of the average position of the peak. The experimental RR scattering condition is fully achieved with an incident wavelength of 266 nm, but there is another more intense absorption maximum at about 205 nm (Figure 5). Enhancement factors computed with respect to the Raman cross-sections at 1064 nm.

Vibration	Position	StdDev	Raman	RR	RR ^1^	Enhancement Factor	
	cm^−1^	cm^−1^	@1064 nm	@266 nm	@210 nm	@266 nm	@210 nm
$\rho_{{\mathrm{CH}}_{3},M1}$	1050/1049	5	5.07 × 10^−10^	0.0023	0.0084	4.54 × 10^6^	1.66 × 10^7^
$\rho_{{\mathrm{CH}}_{3},M3}$	1101/1097	7	9.73 × 10^−10^	0.0026	0.0164	2.67 × 10^6^	1.69 × 10^7^
$\nu_{\mathrm{rings}}$	1308/1313	6	2.13 × 10^−9^	0.0026	0.0120	1.22 × 10^6^	5.63 × 10^6^
$\nu_{i-\mathrm{ring},2}$	1360/1362	4	3.71 × 10^−9^	0.0011	0.0383	2.96 × 10^5^	1.03 × 10^7^
$\nu_{i-\mathrm{ring},1}$	1396/1394	4	4.15 × 10^−9^	0.0034	0.0074	8.19 × 10^5^	1.78 × 10^6^
$\delta_{C8-H}$	1455, 1469/1469	7	1.38 × 10^−9^	0.0014	0.0110	1.01 × 10^6^	7.97 × 10^6^
$\delta_{\mathrm{CM}}$	1517, 1538/1536	10	1.58 × 10^−9^	0.0013	0.0104	8.23 × 10^5^	6.58 × 10^6^
$\nu_{C-C}$	1583/1583	4	5.90 × 10^−10^	0.0002	0.0140	3.39 × 10^5^	2.37 × 10^7^
$\nu_{C=C}$ + $\nu_{C=N}$	1618/1624	6	2.12 × 10^−9^	0.0003	0.0265	1.42 × 10^5^	1.25 × 10^7^

^1^ Among the $\omega_{0}$ list used to compute RR cross-sections, this value is the closest one to the absorption maximum at 205 nm.

## Data Availability

The original contributions presented in the study are included in the article/Supplementary Material, further inquiries can be directed to the corresponding authors.

## Supplementary Materials

The following supporting information can be downloaded at: https://www.mdpi.com/article/10.3390/molecules29133035/s1, NBO and QTAIM analyses; Additional plot concerning UV-Vis spectra; Analysis of electronic transitions; Raman and RR spectra in the gas phase and PCM; Stick-spectra; Effect of specific excited states; Shift vector; RREP; Convergence plots.

## Abbreviations

The following abbreviations are used in this manuscript:

(UV)RR	(Ultra–Violet) Resonance Raman
QM	Quantum Mechanics
FQ	Fluctuating Charges
RREP	Resonance Raman Excitation Profile
MD	Molecular Dynamics
SERS	Surface-Enhanced Raman Scattering
RDF	Radial Distribution Function
NBO	Natural Bond Orbitals
BCP	Bond Critical Point
QTAIM	Quantum Theory of Atoms in Molecules
FWHM	Full Width at Half Maximum
PCM	Polarizable Continuum Model
