# Supplementary material for: The Role of Hydrogen Bonding in the Raman Spectral Signals of Caffeine in Aqueous Solution
Source: Molecules. 2024 Jun 26;29(13):3035. doi: 10.3390/molecules29133035 (PMC11243038; doi:10.3390/molecules29133035)
Supplement: Supplementary file 1 [file molecules-29-03035-s001.zip › molecules-3071093-supplementary.pdf]

The Role of Hydrogen Bonding in the Raman Spectral Signals of Caffeine in Aqueous Solution

Sara Gómez and Chiara Cappelli

Scuola Normale Superiore, Classe di Scienze, Piazza dei Cavalieri 7, 56126, Pisa, Italy

Supplementary Material

Contents

1 NBO and QTAIM analyses **S2**

2 Additional data concerning UV-Vis **S4**

3 Additional plots concerning Raman and RR spectroscopies **S6**

4 Convergence plots **S11**

1 NBO and QTAIM analyses

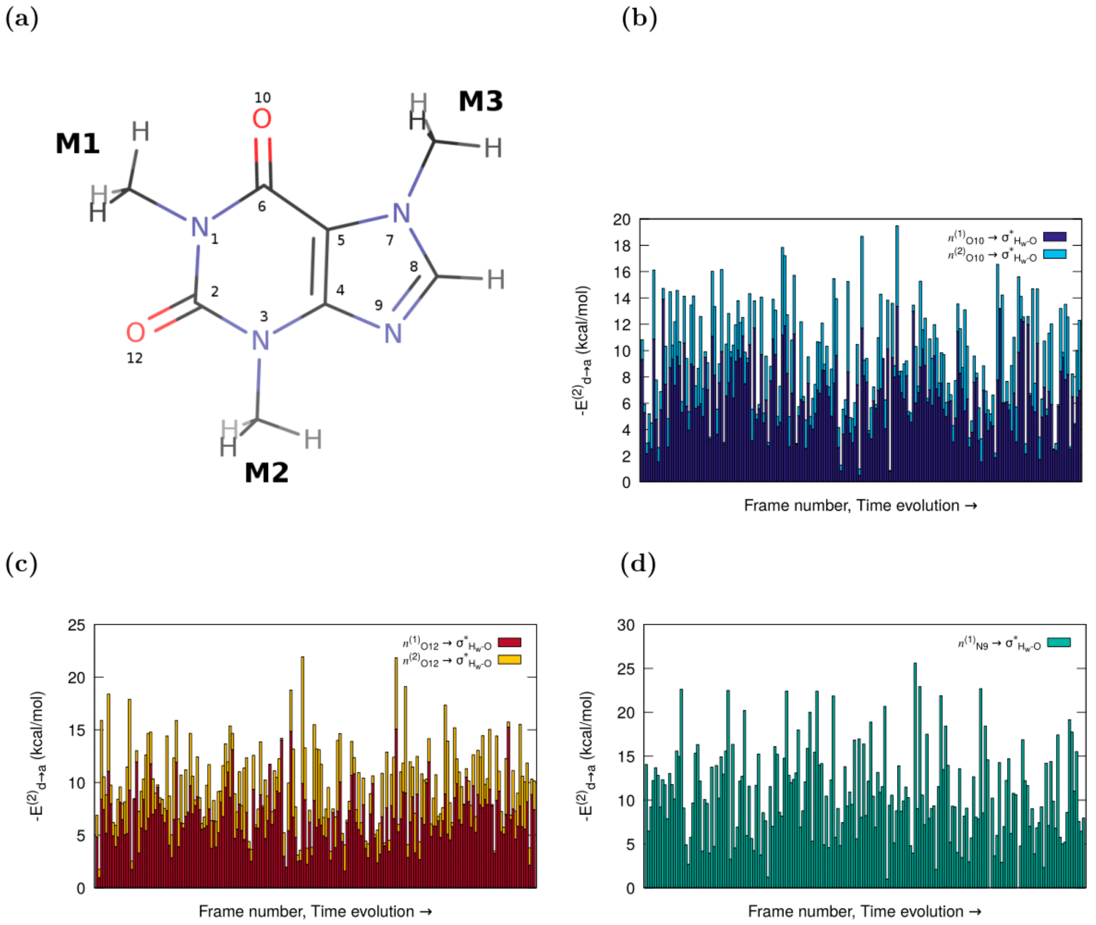

Figure S1: Caffeine labeling (panel a) and evolution of the stabilization energies associated to the $n_{Oc} \rightarrow \sigma^{*}_{H_w-O}$ Oc = O10, O12 charge transfers, panels b) and c), respectively, and $n_{N9} \rightarrow \sigma^{*}_{H_w-O}$ charge transfer, panel d), along the entire MD simulation. For the stacked plots, the largest $\left|E^{(2)}_{d\rightarrow a}\right|$ is chosen.

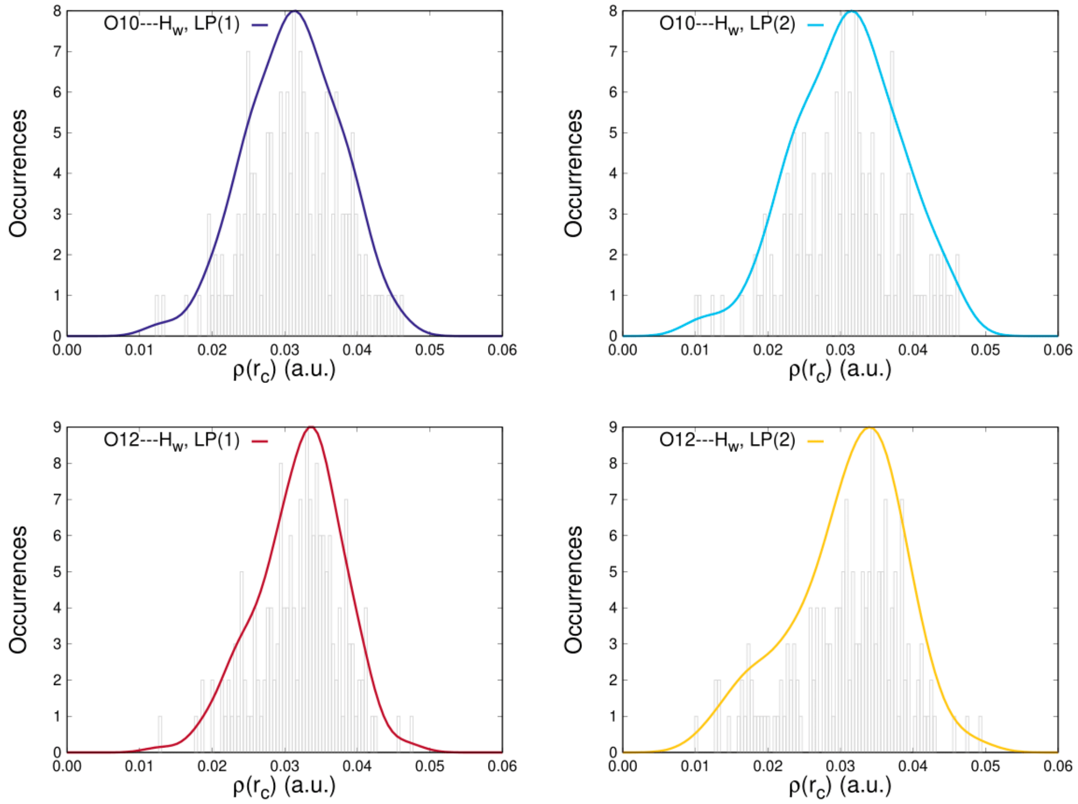

Figure S2: Distribution of the electron densities at the bond critical points for the O10$\cdots$ H_w and O12$\cdots$ H_w intermolecular contacts between caffeine and water molecules along the MD trajectory. Interactions with each lone pair (LP) of each oxygen atom are considered.

2 Additional data concerning UV-Vis

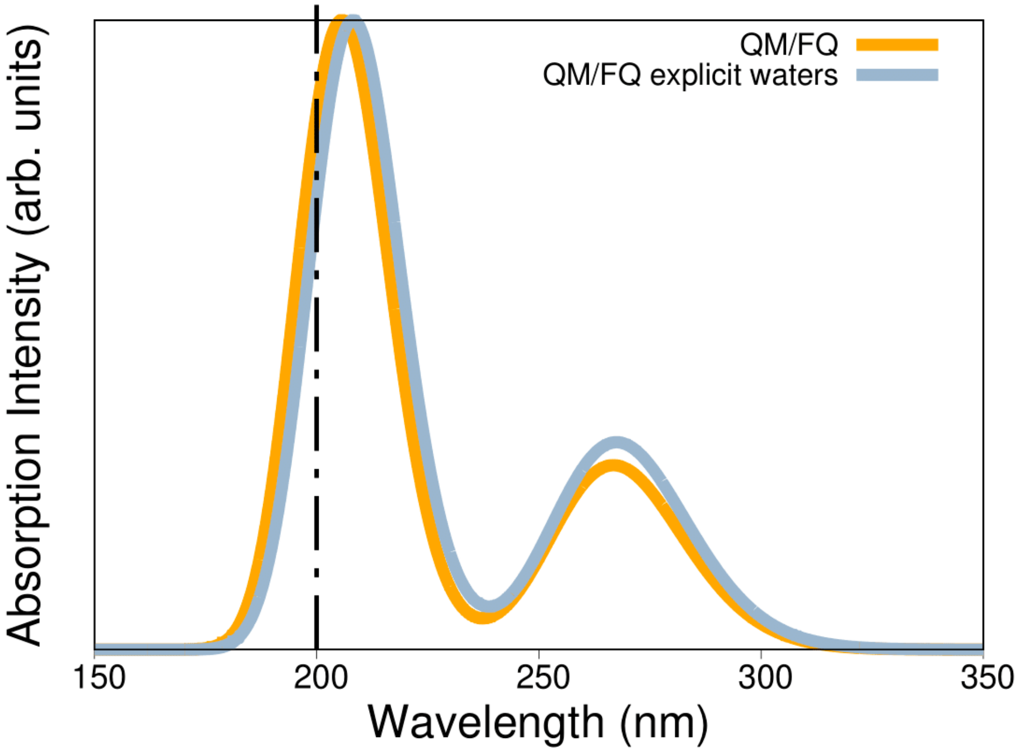

Figure S3: Effect of the solute optimization and of the inclusion of explicit water molecules on the final UV–Vis spectra of Caffeine in water. 200 structures were considered for calculating the absorption spectra at the QM/FQ level, with B3LYP/6–311++G(*d*, *p*).

Table S1: Vertical excitation energy VEE (eV), oscillator strength (*f*) and main Molecular Orbital transitions of the first 10 excited states of Caffeine in water calculated at the QM/FQ level, with B3LYP/6–311++G(*d, p*) using one of the snapshots extracted from the MD. H and L stand for HOMO and LUMO, respectively.

State	VEE	*f*	Transition	Coefficient
S_1	4.5799	0.1789	H → L	0.48
S_2	5.0891	0.0003	H-2 → L	0.42
			H-1 → L	0.04
S_3	5.5107	0.0154	H-1 → L	0.25
			H → L+1	0.15
S_4	5.8315	0.0086	H → L+2	0.45
S_5	5.9312	0.4162	H → L+1	0.31
			H-1 → L	0.12
S_6	6.0859	0.0751	H-3 → L	0.36
			H → L+3	0.06
S_7	6.1759	0.1682	H → L+3	0.37
			H-3 → L	0.09
S_8	6.2248	0.0054	H-4 → L	0.41
S_9	6.2497	0.0223	H → L+4	0.46
S_{10}	6.4300	0.0015	H-2 → L+1	0.33
			H-4 → L+1	0.10

3 Additional plots concerning Raman and RR spectroscopies

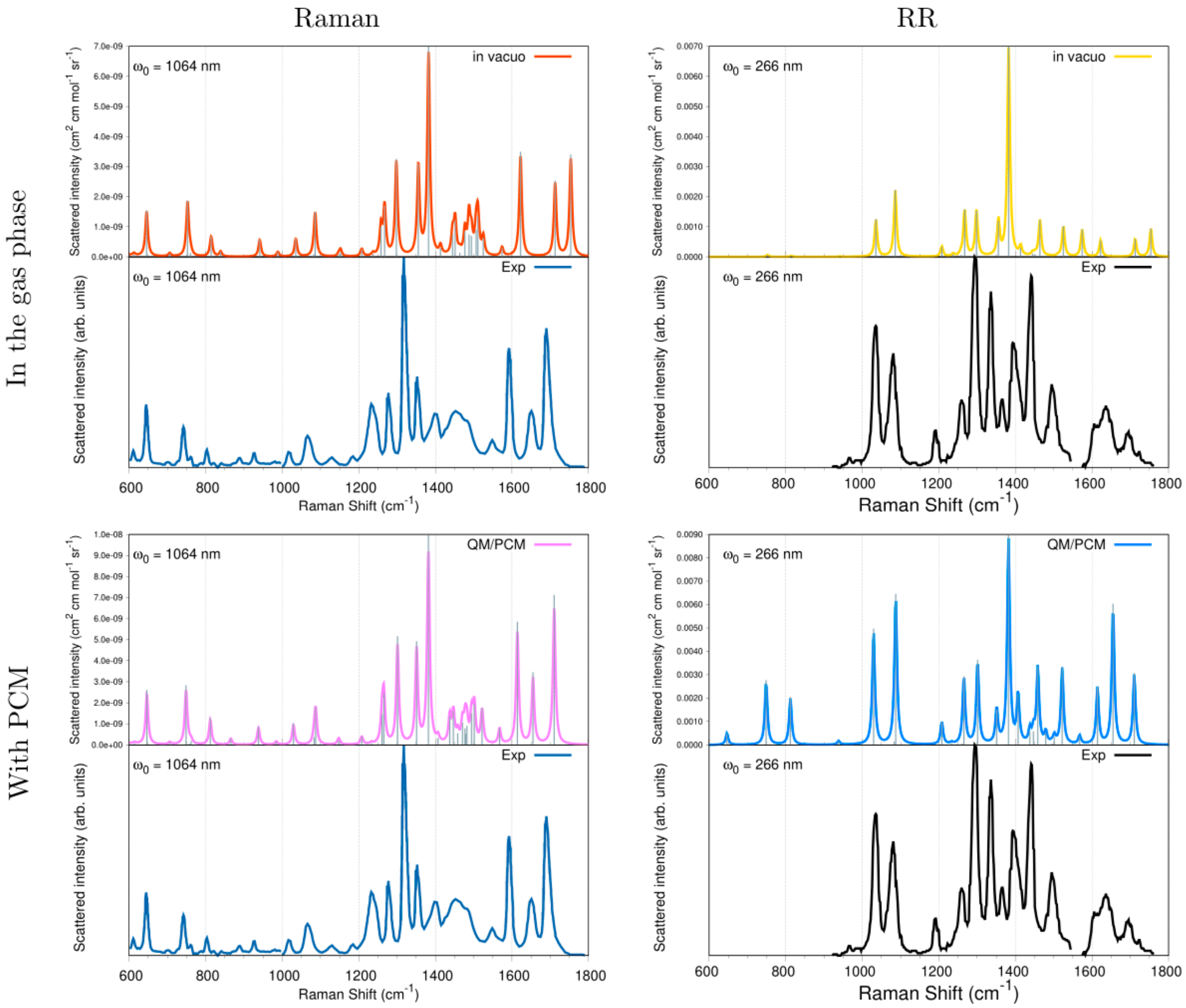

Figure S4: Raman (left) and RR (right) spectra of caffeine computed in the gas phase and in solution by means of PCM. Sticks for spontaneous Raman and RR are convoluted with Lorentzian profiles and FWHM values of 8 cm^{-1}

(a)

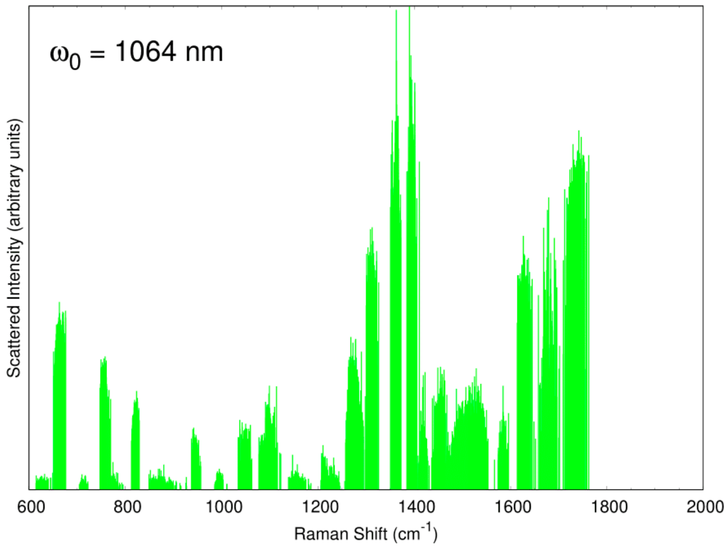

(b)

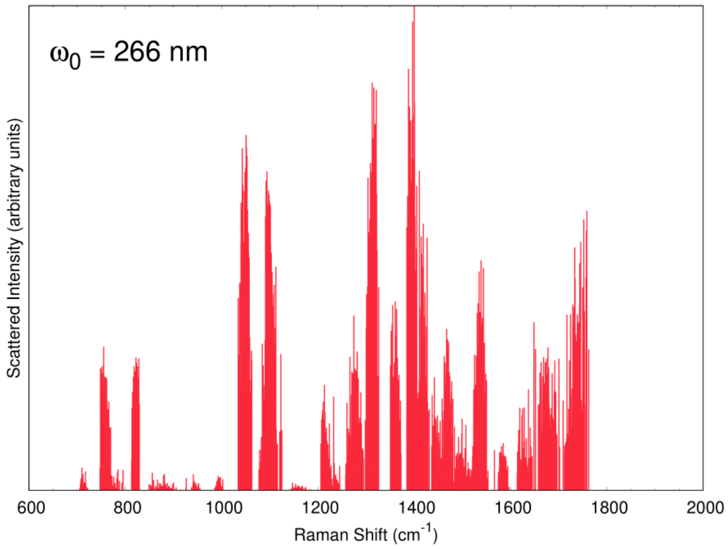

Figure S5: (a) Raman and (b) RR stick spectra of caffeine in aqueous solution. 200 frames are included in each case.

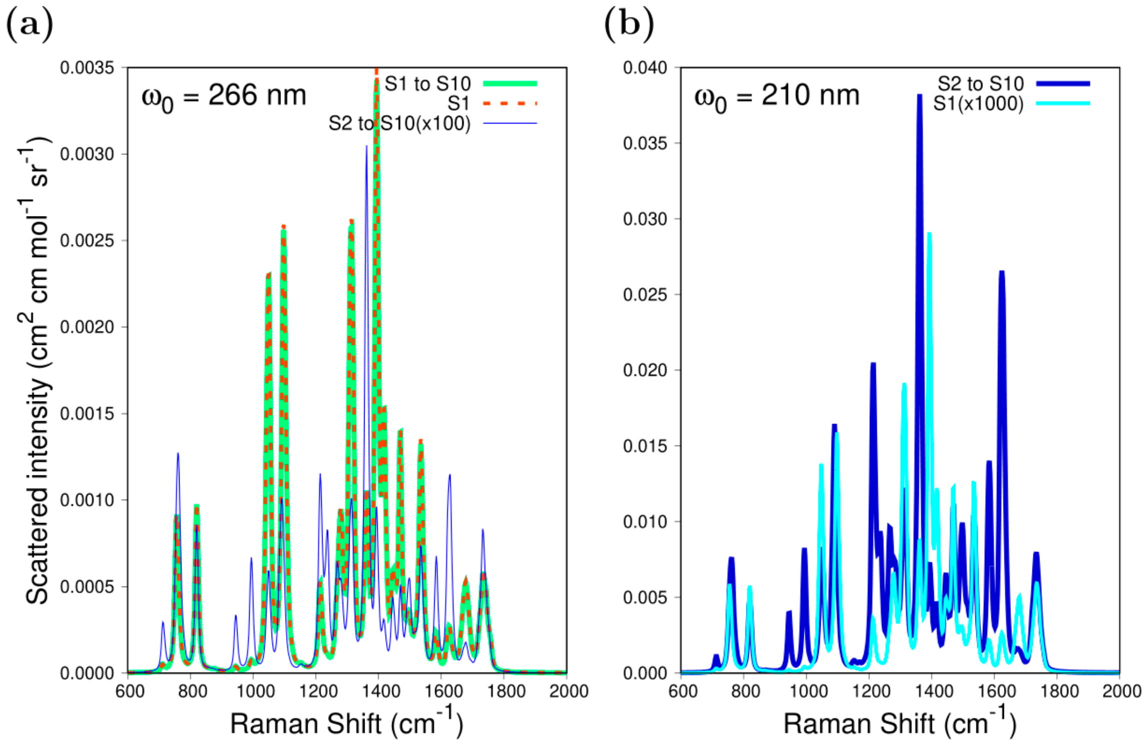

Figure S6: Effect of specific excited states on the selectivity and intensity of the QM/FQ in Resonance Raman spectra of caffeine in aqueous solution.

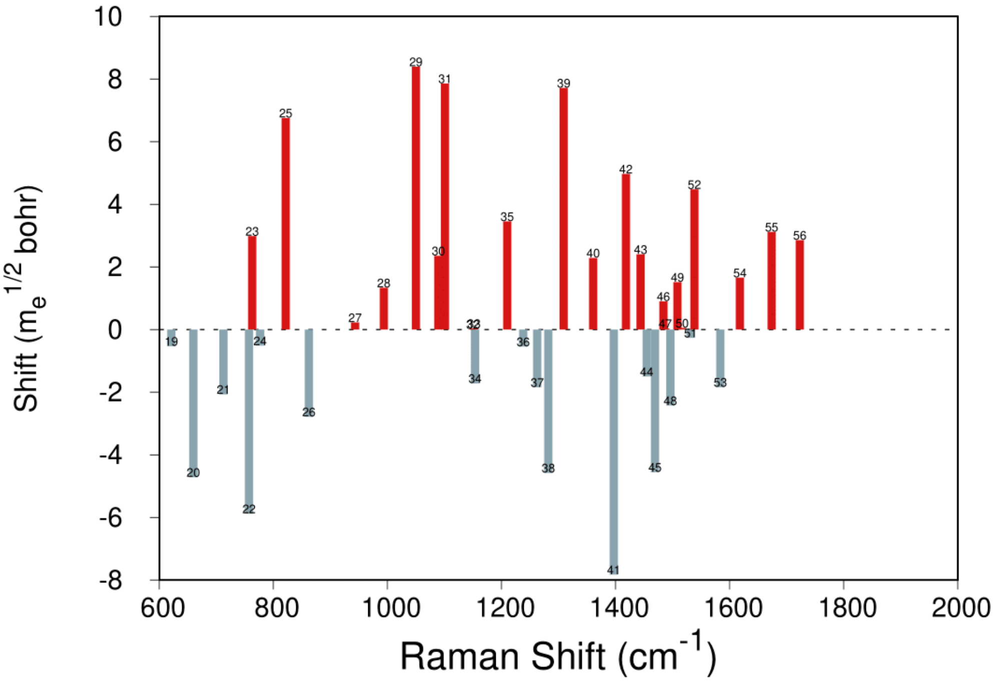

Figure S7: Graphical representation of the shift vector for the $\pi \rightarrow \pi^*$ transition ($S_0 \rightarrow S_1$) of caffeine in water. Data taken from a single snapshot. Red and gray bars are associated with positive and negative values of the shift vector, respectively. Note that for each normal mode j, there is a relationship between the shift vector and the so-called "dimensionless displacement" $\Delta_j = K_j \left(\frac{\omega_j^g}{\hbar}\right)^{\frac{1}{2}}$ with g standing for the ground electronic state. In the Vertical Gradient model, displacements are obtained from the excited state gradient at the ground-state geometry.

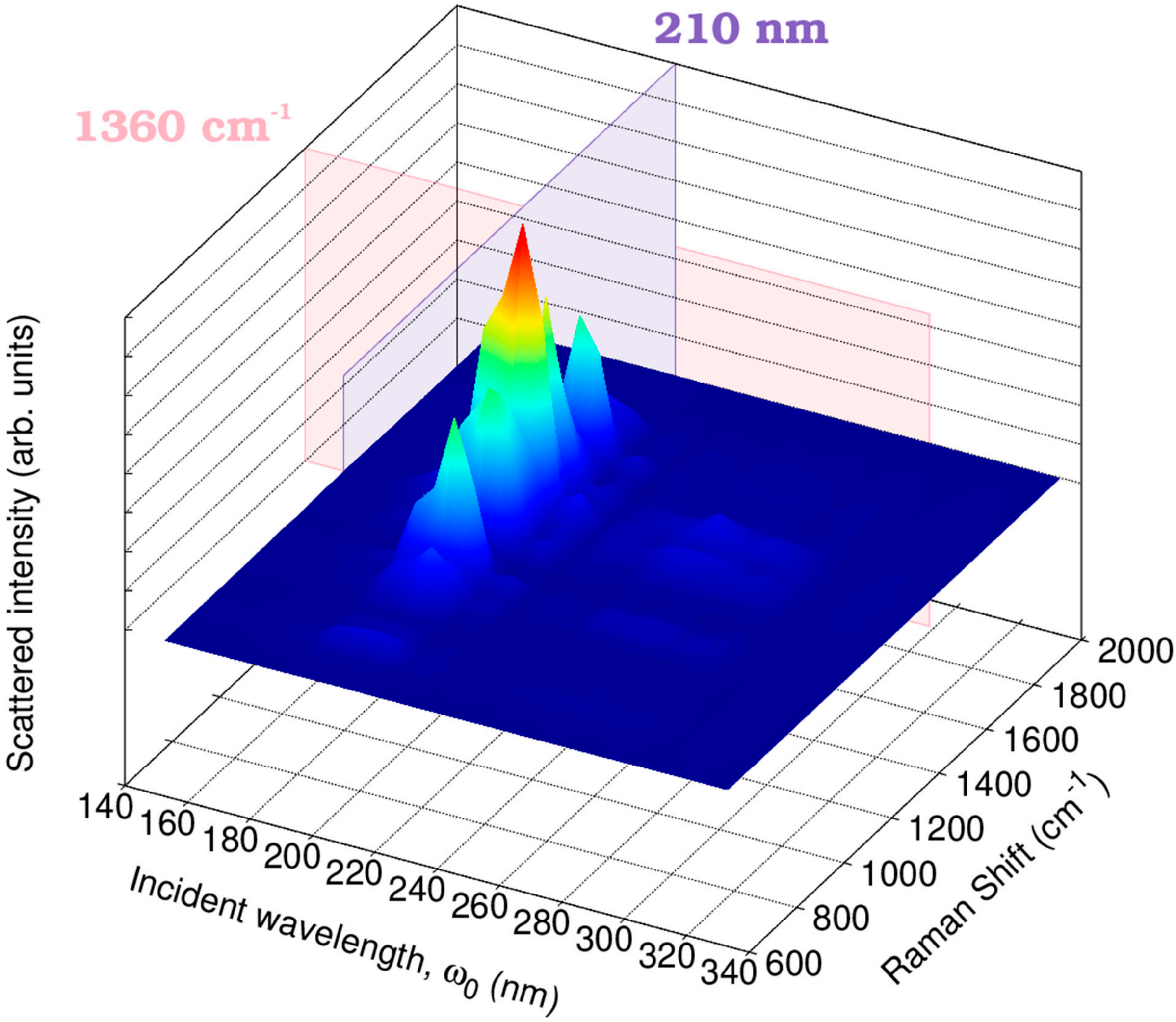

Figure S8: Calculated QM/FQ Resonance Raman Excitation Profiles (RREP) of caffeine in aqueous solution. 200 structures were considered to achieve convergence at the QM/FQ level, with B3LYP/6–311++G(*d, p*). Planes cutting at 210 nm and 1360 cm^{-1} are also included for visualization purposes.

4 Convergence plots

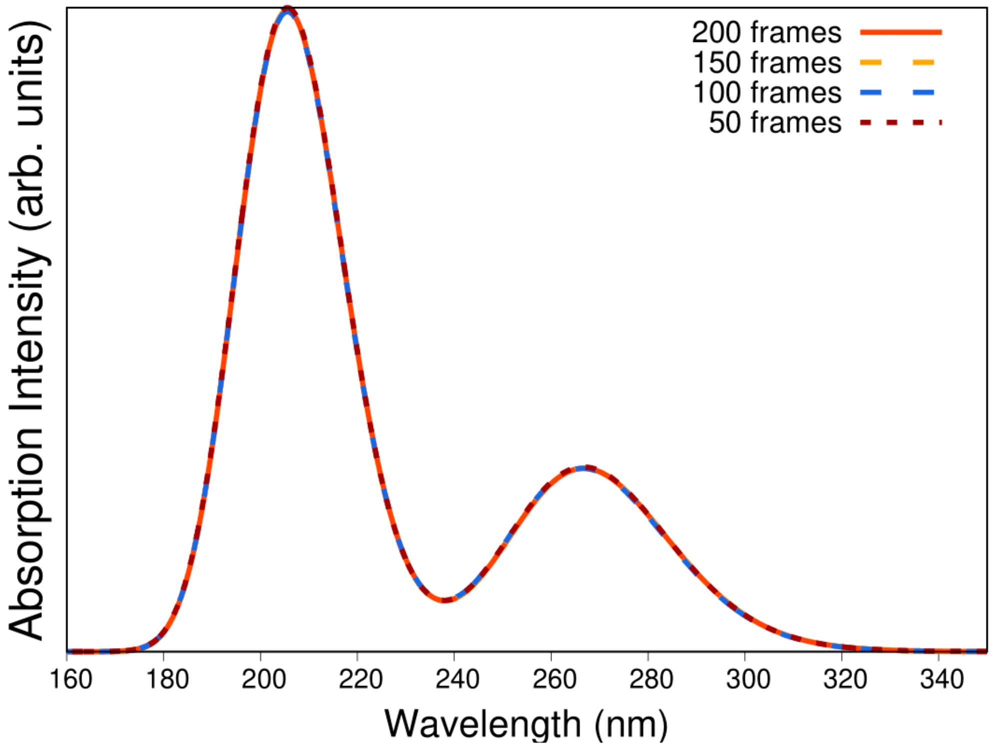

Figure S9: QM/FQ absorption spectra of caffeine in aqueous solution obtained by averaging an increasing number of snapshots.

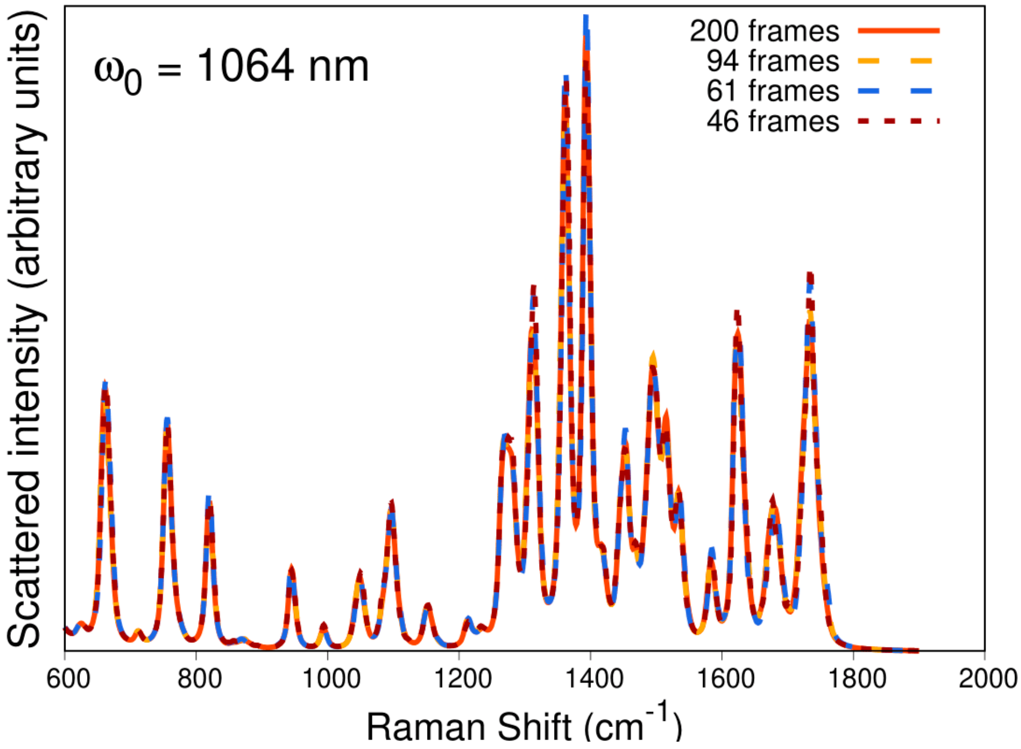

Figure S10: QM/FQ Raman spectra of caffeine in aqueous solution obtained by averaging an increasing number of snapshots.

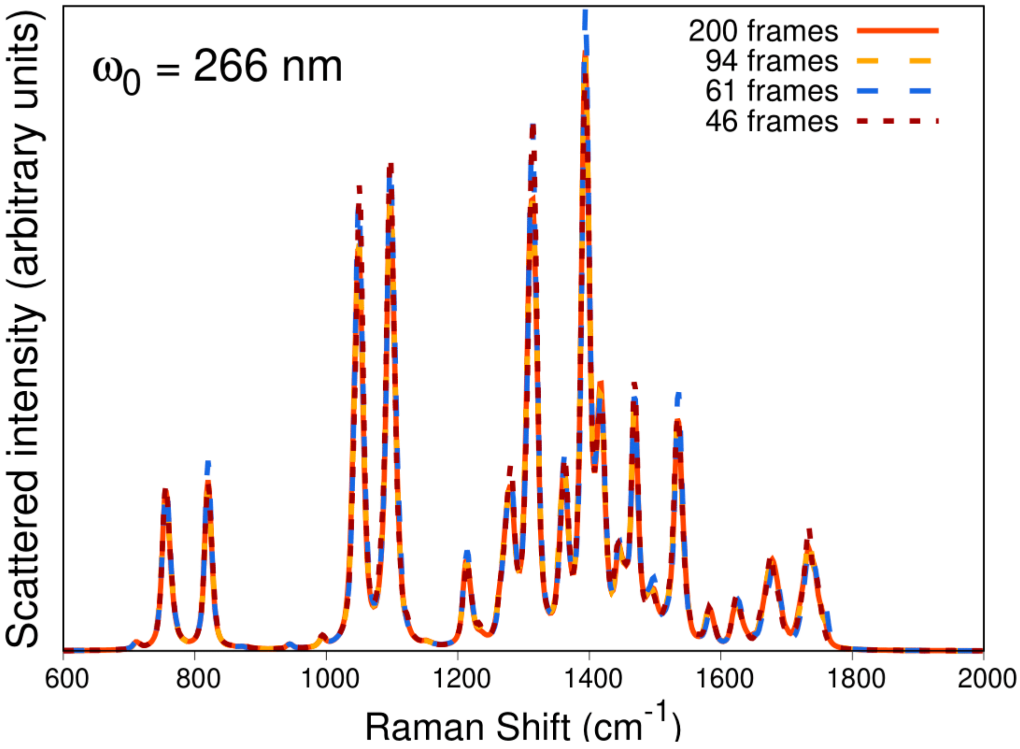

Figure S11: QM/FQ Resonance Raman spectra of caffeine in aqueous solution obtained by averaging an increasing number of snapshots.
